# Supplementary material for: Age and Sex Specific Prevalence of Clinical and Screen-Detected Atrial Fibrillation in Hospitalized Patients
Source: J Clin Med. 2021 Oct 22;10(21):4871. doi: 10.3390/jcm10214871 (PMC8584962; doi:10.3390/jcm10214871)
Supplement: Supplementary file 1 [file jcm-10-04871-s001.zip › jcm-1413565-supplementary.pdf]

Table S1. Circumstances of atrial fibrillation diagnosis outside of a 7-day Holter ECG.

Case #	Number of 7-day Holter ECGs Performed	Circumstances of Atrial Fibrillation Diagnosis
1	0	Diagnosis of atrial fibrillation 3 days after study inclusion but before hospital discharge
17	1	Diagnosis of atrial fibrillation in an exercise test during cardiac rehabilitation
18	1	Diagnosis of atrial fibrillation during an emergency unit visit because of dyspnea
19	1	Diagnosis of atrial fibrillation during an emergency unit visit because of dizziness and nausea
20	1	Diagnosis of complete AV block during the first 7-day Holter ECG with consecutive pacemaker implantation. Diagnosis of atrial fibrillation in the first pacemaker visit after implantation.
21	1	Diagnosis of atrial fibrillation during the second study visit in the 12-lead ECG
27	2	Diagnosis of atrial fibrillation during an emergency unit visit because of angina pectoris
28	2	Diagnosis of complete AV block during the second 7-day Holter ECG with consecutive pacemaker implantation. Diagnosis of atrial fibrillation in the first pacemaker visit after implantation.
38	3	Diagnosis of atrial fibrillation during a hospitalization 2 weeks after the last 7-day Holter ECG

Table S2. Atrial fibrillation diagnosis during 7-day Holter ECGs.

	All Positive 7d-Holter ECGs	First 7d-Holter ECG	Second 7d-Holter ECG	Third 7d-Holter ECG	*P* Value
Atrial fibrillation or atrial flutter	29	15	5	9	0.367
Atrial fibrillation	27 (93%)	13 (87%)	5 (100%)	9 (100%)	0.367
Atrial flutter	-	-	-	-	-
Both	2 (7%)	2 (13%)	-	-	0.367
Number of atrial fibrillation episodes	2 (1; 6)	2 (1; 8)	2 (1; 8)	1 (1; 3)	0.351
Longest atrial fibrillation episode, min.	163 (29; 520)	163 (10; 743)	431 (171; 651)	83 (37; 458)	0.489
Total atrial fibrillation episodes, min.	198 (41; 544)	170 (20; 1119)	431 (178; 677)	124 (41; 459)	0.508
Appropriate signal recording to diagnosis, hours	32 (4; 104)	32 (4; 68)	97 (25; 134)	14 (2; 128)	0.562

Table S3. Prevalence estimates of screen-detected atrial fibrillation in percentages with 95% Normal-Wald confidence intervals.

Sex	Age	% (95%-CI Normal-Wald)
Overall	All ages	4.94 (3.30–6.58)
Male	All ages	5.48 (3.18–7.78)
Female	All ages	3.98 (1.99–5.98)
Overall	65–69	4.06 (1.12–7.01)
	70–74	5.17 (1.86–8.48)
	75–79	5.38 (1.97–8.79)
	80–85	5.45 (2.12–8.78)
Male	65–69	3.97 (0.04–7.90)
	70–74	5.82 (1.19–10.44)
	75–79	6.20 (1.30–11.09)
	80–84	6.86 (1.83–11.90)
Female	65–69	4.25 (0.10–8.41)
	70–74	3.90 (0.06–7.74)
	75–79	3.92 (0.06–7.78)
	80–84	3.82 (−0.49–8.12)
Male	<75	4.90 (1.88–7.91)
	≥75	6.44 (2.85–10.02)
Female	<75	4.07 (1.27–6.88)
	≥75	3.87 (1.02–6.72)

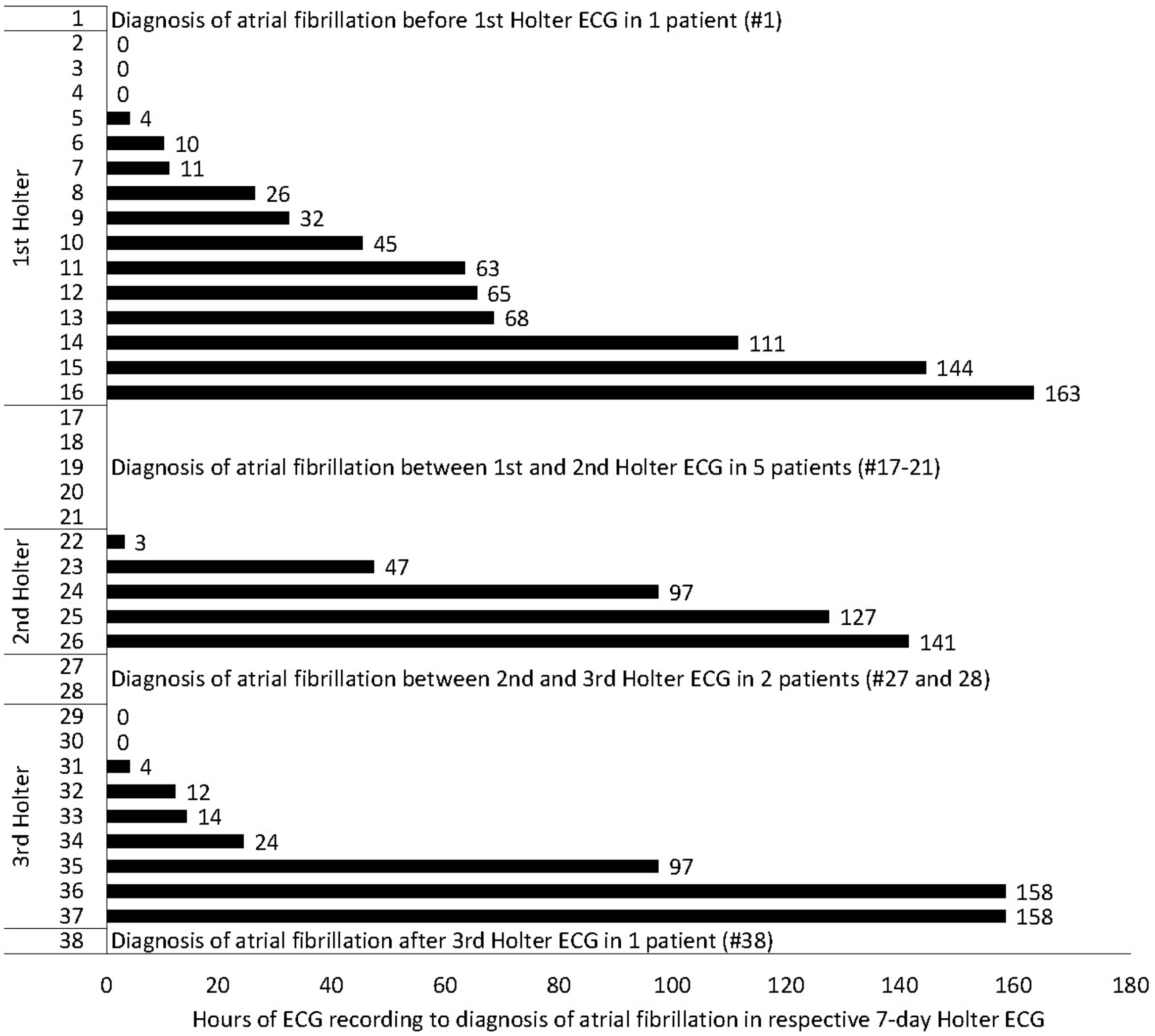

Figure S1. Modality and time point of diagnosis of atrial fibrillation for the 38 cases with screen-detected atrial fibrillation. Y-axis: cases #1-38; X-axis: hours of ECG recording to diagnosis of atrial fibrillation in the respective 7-day Holter ECGs for the 29 cases with atrial fibrillation diagnosis in a 7-day Holter ECG.
